# Systematic Investigation of TCP Gene Family: Genome-Wide Identification and Light-Regulated Gene Expression Analysis in Pepino (*Solanum Muricatum*)

**DOI:** 10.3390/cells12071015

**Published:** 2023-03-26

**Authors:** Cheng Si, Deli Zhan, Lihui Wang, Xuemei Sun, Qiwen Zhong, Shipeng Yang

**Affiliations:** 1 Laboratory for Research and Utilization of Germplasm Resources in Qinghai Tibet Plateau, Agriculture and Forestry Sciences Institute of Qinghai University, Xining 810016, China; qhdxsc@163.com (C.S.); zdl19981118@163.com (D.Z.); wanglihui@qhu.edu.cn (L.W.); 2007990021@qhu.edu.cn (X.S.); 2 College of Agriculture and Animal Husbandry, Qinghai University, Xining 810016, China; 3 College of Life Sciences, Northwest A&F University, Yangling 712100, China

**Keywords:** TCP gene family, *S. muricatum*, RNA-seq, light response

## Abstract

Plant-specific transcription factors such as the TCP family play crucial roles in light responses and lateral branching. The commercial development of *S. muricatum* has been influenced by the ease with which its lateral branches can be germinated, especially under greenhouse cultivation during the winter with supplemented LED light. The present study examined the TCP family genes in *S. muricatum* using bioinformatics analysis (whole-genome sequencing and RNA-seq) to explore the response of this family to different light treatments. Forty-one TCP genes were identified through a genome-wide search; phylogenetic analysis revealed that the CYC/TB1, CIN and Class I subclusters contained 16 SmTCP, 11 SmTCP and 14 SmTCP proteins, respectively. Structural and conserved sequence analysis of SmTCPs indicated that the motifs in the same subcluster were highly similar in structure and the gene structure of SmTCPs was simpler than that in *Arabidopsis thaliana;* 40 of the 41 SmTCPs were localized to 12 chromosomes. In *S. muricatum*, 17 tandem repeat sequences and 17 pairs of SmTCP genes were found. We identified eight TCPs that were significantly differentially expressed (DETCPs) under blue light (B) and red light (R), using RNA-seq. The regulatory network of eight DETCPs was preliminarily constructed. All three subclusters responded to red and blue light treatment. To explore the implications of regulatory TCPs in different light treatments for each species, the TCP regulatory gene networks and GO annotations for *A. thaliana* and *S. muricatum* were compared. The regulatory mechanisms suggest that the signaling pathways downstream of the TCPs may be partially conserved between the two species. In addition to the response to light, functional regulation was mostly enriched with auxin response, hypocotyl elongation, and lateral branch genesis. In summary, our findings provide a basis for further analysis of the TCP gene family in other crops and broaden the functional insights into TCP genes regarding light responses.

## 1. Introduction

The *Solanum* genus includes important food crops such as *Solanum lycopersicum* (tomato), *Solanum tuberosum* (potato), and *Solanum muricatum* (pepino), which are members of the *Solanaceae* family. *Solanum* is one of the largest angiosperm genera, and it consists of annual and perennial plants in diverse habitats. There are now various and more advanced studies on its genome, molecular biology, etc. [1,2,3]. Compared with important botanical families of horticultural fruit crops, the *Solanaceae* family includes several economically important vegetable crops, some of which were described above, with this species representing an important component of human diets worldwide; however, *S. muricatum* is one of the few new fruit crops of the *Solanaceae* family that have been successfully adopted by international markets in recent years. *S. muricatum* originates from the Andes and belongs to the genus *Solanum* in the *Solanaceae* family. It is an evergreen shrub grown mainly in South America, New Zealand, and Spain for its aromatic and juicy fruit [4]. Its fruits have many benefits, and they have traditionally been used as a remedy for diabetes mellitus, hypertension, and sprue [5]. *S. muricatum* also has the potential to act as a tumor repressor [6]. In China, *S. muricatum* has been cultivated in high-altitude regions such as Qinghai, Gansu, and Yunnan at elevations of up to 1500 m above sea level [7]. *S. muricatum*, in particular, is typically produced in greenhouses during cold periods of the year at the northern latitudes of China. During this time, the plants are cultivated under the artificial high-light conditions in greenhouses, which are implemented in response to insufficient sunshine. As intensive cultivation structures make it possible to obtain the maximum possible fruit yield, the planting density affects shoot branching in *S. muricatum*. Its lateral branches are clustered and germinate vigorously [8]. It is apparent that the biological characteristics of easy germination in the lateral branches influence its commercial development. Presently, lateral branch pruning is a time- and labor-intensive process that requires substantial resources [9]. In accordance with our previous study, correlation analysis revealed that the gene expression patterns correlated with lateral branching under various light treatments in *S. muricatum* [10]. Significant differential expression can be observed for the TCP transcription factor TEOSINTE BRANCHED under different light treatments. Therefore, we initially hypothesized that the plants can distinguish between different light conditions, selecting patterns that reach equilibrium during development. The same TCP regulatory mechanisms in different light treatments might exist for model species such as *Arabidopsis thaliana* [10].

Plant transcription factors (TFs) play important roles in diverse biological processes. TFs are DNA-binding proteins that bind to specific cis-elements and directly regulate the transcription of DNA to mRNA [11,12]. The teosinte branched1/cycloidea/proliferating cell factor (TCP) family comprises plant-specific TFs that are primarily expressed in the fast-growing tissues or organs. TCPs play significant roles in cell differentiation and development [13]. This protein family is predominantly represented by four members, namely TEOSINTE BRANCHED1 (TB1), CYCLOIDEA (CYC), PROLIFERATING CELL NUCLEAR ANTIGEN FACTOR1 (PCF1), and PCF2. The TCP genes encode conserved sequences of about 60 amino acids designated as the TCP domain. A basic helix–loop–helix (bHLH) structure is formed from the TCP domain [14]. This domain may be important for the activation or repression of transcription and is involved in protein–protein interactions. TCP proteins can be classified into two major classes based on the conserved structural domain sequence and their phylogenetic relationships: class I (also known as PCF or TCP-P class) and class II (also known as TCP-C class) [15]. In angiosperms, class II TCPs can be further categorized into two branches based on the differences within the TCP domain: CYC/TB1 and CIN (CINCINNATA) [16,17]. The CYC/TB1 subclade originated after CIN, and its members are primarily involved in regulating shoot branching and apical dominance [18]. The TEOSINTE BRANCHED1 (TB1) gene regulates apical dominance in maize (*Zea mays* L.) [19]. In addition, it inhibits lateral branch growth and male flower formation [20,21]. The CYC subclade is represented by CYC and TB1, and CYC from snapdragon (*Antirrhinum majus*), which regulates flower development [22]. PROLIFERATING CELL FACTORS1 (PCF1) and 2 (PCF2) are essential for meristem-specific expression and function as negative regulatory elements for lateral branch development in rice [23,24]. A few TCP genes are also involved in photoresponse (response to light). Members of the TEOSINTE BRANCHED1, CYCLOIDEA, and PCF (TCP) family are involved in photomorphogenesis [25]. TCP17 may regulate the shade-induced hypocotyl elongation rate in *A. thaliana* [26]. TCP2 interacts with the cryptochromes and is involved in blue light-stimulated photomorphogenesis in *A. thaliana* [27]. To date, TCP genes have been identified in several plant species, including 24 family members in *A. thaliana* [28], 23 in *Oryza sativa*, 30 in *S. lycopersicum* [29], and 31 in *S. tuberosum* [30]. However, systematic investigation of the TCP gene family in *S. muricatum* has not been reported to date.

Our group discovered a differential response of pepino to red and blue light in lateral branch genesis in our preliminary study. Overall, red light facilitated internode elongation and inhibited lateral branching, while the lateral branches elongated under blue light, in which *BRC2* played a major role [10]. We reviewed a large amount of research and finally located TCP in lateral branch development and light quality response. Unlike for other crops, few studies have been conducted on *S. muricatum;* the roles of TCPs in light responses in *S. muricatum* remain largely unknown [31,32,33]. Meanwhile, our study unraveled the response of TCPs to light and the regulatory mechanisms in *S. muricatum* and compared their co-expression network in *A. thaliana*. Overall, our findings provide a roadmap for further analysis of the TCP gene family in other crops and broaden the functional insights into TCP genes regarding light responses.

## 2. Materials and Methods

### 2.1. Identification, Basic Characterization and Chromosome Location of SmTCPs

The HMM model was downloaded from the Pfam database (http://pfam.xfam.org (accessed on 6 February 2022)), and we searched the whole genome of *S. muricatum* using TCP (PF03634) as a query with a threshold of 1.2 × 10^−28^. Subsequently, duplicates were removed from the retrieved genes using the online sites SMART (http://smart.emblheidelberg.de/ (accessed on 6 February 2022)) and NCBI (https://www.ncbi.nlm.nih.gov/ (accessed on 6 February 2022)). The gene, CDS, and protein sequences, as well as gene structure and chromosomal location information of TCP family members, were extracted from the whole-genome database involving *S. muricatum* TCPs (SmTCPs) (http://bigd.big.ac.cn/gsa/ (accessed on 15 February 2022) Accession numbers CRA005032 and CRA005096). Finally, the positions of TCP genes on the respective chromosomes were mapped using the MapInspect software(v1.0) [34].

### 2.2. Phylogenetic Analysis of SmTCPs

A neighbor-joining (NJ) phylogenetic tree was built to show the evolutionary relationship between AtTCPs, StTCPs, SlTCPs, and SmTCPs. The protein sequences of SmTCP family members were subjected to ClustalW multiple alignments. In addition, the phylogenetic tree was constructed following the NJ method using MEGA7.0 with 1000 bootstrap values.

### 2.3. Conserved Motifs and Gene Structure Analysis

The gene structures were determined using the CDS and DNA sequences of SmTCP genes and visualized with the Gene Structure Display Server (http://gsds.gao-lab.org/ (accessed on 20 February 2022)). In order to identify the conserved motifs among all of the SmTCP genes, their protein sequences were subjected to the online software MEME (http://meme-suite.org/tools/meme/ (accessed on 20 February 2022)) using default parameters, with the exception of the number of motifs, which was set to 20. 

### 2.4. Promoter Region Cis-Acting Element Analysis 

We extracted the upstream 1500 bp sequence of each SmTCP gene for promoter analysis. The cis-acting elements present in SmTCP promoters were predicted using the online prediction software PlantCare [35]. The prediction results and enriched cis-acting elements were visualized and analyzed using the Simple Biosequenceviewer function on TBtools [36]. Intraspecific and interspecific synteny relationships among TCP genes were determined using MCScanX [37].

### 2.5. Data mining of A. thaliana Gene Regulatory Network 

We identified the target genes for the *A. thaliana* TCP (AtTCP) gene family using the Integrated Gene Regulatory Network (iGRN) database (http://bioinformatics.psb.ugent.be/webtools/iGRN/ (accessed on 6 February 2022)), looking for scores ≥ 0.80. In addition, target genes were classified into downstream genes (regulated by TCP family genes) and upstream genes (regulated TCP family genes). Finally, an interaction network combining TCP family members and their target genes was constructed using Gephi (v0.9.2) and ForceAtlas2 (https://gephi.org/ (accessed on 6 February 2022)) [38].

### 2.6. RNA Extraction and Transcriptome Sequencing 

We used the *S. muricatum* SRF (sweet round fruit) cultivar as the plant material, provided by the Institute of Horticulture, Qinghai Academy of Agriculture Forestry Science (101°45′08″ E, 36°43′32″ N). The light quality was tested in a phytotron. Each layer of the culture holder was set up with full spectrum, red and blue LED lights. The full spectrum light was used as a control, adjusting the light intensity to 100 ± 10 μmol m^–2^ s^–1^. The illumination time was 14 h/d. *S. muricatum* cultivation, sample processing and collection were performed as previously described [10]. 

The total RNA was extracted by using the TaKaRa MiniBEST Plant RNA Extraction Kit (TaKaRa, Tokyo, Japan) from the bud base of cutting seedlings of *S. muricatum* treated with different light qualities—blue light(B), red light (R) and full spectrum (F)—for 10 d (B1, R1, F1), 20 d, and 30 d (denoted as B1, B2, B3, R1, R2, R3, F1, F2, and F3, respectively) (Figure S1); Paired-end cDNA sequencing libraries were prepared with the IlluminaTruSeq Stranded Total RNA Library Preparation Kit following the manufacturer’s protocol. Total RNA was purified and subsequently identified and quantified. The RNA was quantified using the Qubit RNA Assay Kit in a Qubit 2.0 Fluorometer (Life Technologies, Carlsbad, CA, United States), after which the integrity of each RNA sample was checked using the RNA Nano 6000 Assay Kit with the Agilent Bioanalyzer 2100 system (Agilent Technologies, Santa Clara, CA, United States). The final cDNA libraries were sequenced on the Illumina NovaSeq 6000 platform by Guangzhou Gene Denovo Biotechnology Co., Ltd. (Guangzhou, China). For each sample, the cDNA library was sequenced. We preprocessed the downstream data to remove joint-containing and poly-N-containing data while filtering out low-quality data to obtain clean reads, and then, we assembled the transcripts with reference to the *S. muricatum* genome. 

### 2.7. Expression Analysis of SmTCP Genes and Accurate Amplification of Candidate Reference Genes

FPKM values of 41 SmTCPs were screened from RNA-seq data to analyze the expression of SmTCP genes. The gplots [39] and pheatmap [40] packages were used to draw the heat map (FPKM values).

We screened for the differentially expressed TCPs (DETCPs) from all DEGs (differentially expressed genes) under B1–R1, B2–R2, and B3–R3, defined as |log2FC|>1 and FDR ≤ 0.05. DETCPs were further analyzed.

We designed gene-specific qRT-PCR primers for DETCPs. The plant materials used for the RT-PCR, to verify the RNA-seq results, were identical to those used for the RNA-seq. RNA was extracted from the bud base of cutting seedlings of *S. muricatum* treated with light of different qualities, with B, R and F light treatment for 10 d, 20 d, and 30 d, and six DETCPs in three subclusters (CYC/TBI, CIN, and Class 1) were randomly selected with reference to expression profiling. The detailed parameters of each primer pair are given in Table S1. We followed a two-step qRT-PCR protocol for cDNA synthesis (FastKing gRNA Dispelling RT SuperMix, KR118, TIANGEN BIOTECH(BEIJING)CO., LTD) and amplification in successive steps (2*Taq PCR MasterMix II, KT211, TIANGEN BIOTECH CO., LTD) to reduce the undesired primer dimer formation using SYBR Green. The RT-PCR data were analyzed by the 2^-ΔΔCt method.

## 3. Results

### 3.1. Identification, Chromosomal Location, and Classification of TCP Family Members in S. muricatum

**Figure 1:**
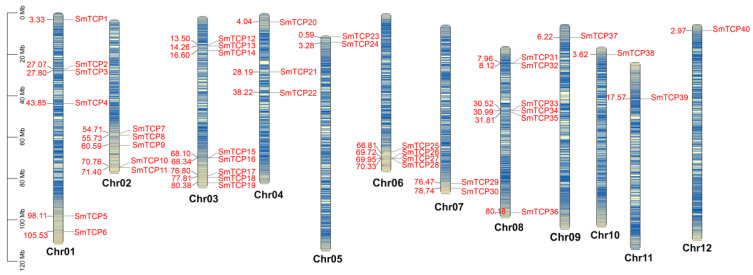
Chromosome density and chromosomal locations of the SmTCPs genes. Note: Chr01-Chr12 indicates 12 chromosomes. Scale bar on the left indicates the chromosome lengths (Mb).

A genome-wide search using the conserved structural domains of the TCP genes identified 41 TCP genes in *S. muricatum* (PF03634). All 41 genes identified through the HMM search were validated for the presence of the TCP structural domain (Table 1). Subsequently, these 41 TCP genes were numbered (Table 1) based on their chromosomal location in *S. muricatum* (Figure 1). Forty of these SmTCPs were distributed across twelve chromosomes, while *SmTCP41* was absent from the annotated chromosomes. A brief analysis of the physicochemical properties revealed that the SmTCPs encoded proteins with amino acids ranging from 165 (*Smu03G006140.1*, *Smu03G006480.1*, *Smu08G008280.1*) to 558 (*Smu10G003890.1*), with an average amino acid number of 315. The molecular weight of the TCP proteins ranged from 18,777.1 to 63,952.9 Da, with an average value of 34,954.2122 Da. The isoelectric point ranged from 4.82 to 11.22, with an average value of 8.609. 

**Figure 2:**
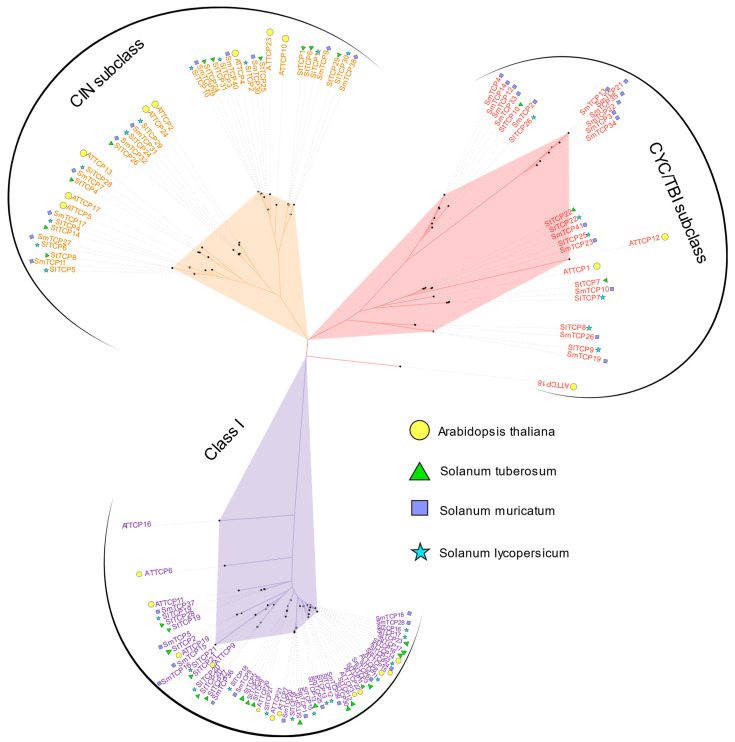
Construction of phylogenetic trees for *S. muricatum*, *S. lycopersicum*, *S. tuberosum* and *A. thaliana*.

Similar to AtTCPs, the SmTCP family members were classified into two subfamilies: Class I and Class II. Class II was further categorized into the CIN and CYC/TB1 subclusters (Figure 2). CIN, CYC/TB1, and Class I contained 11, 16, and 14 TCP members, respectively. The isoelectric points of SmTCPs were different among subclusters. The isoelectric point of each CYC/TB1 member was greater than 7, indicating the predominance of basic amino acids. However, the isoelectric points of the CIN members were either close to seven or less than seven, which highlights their acidic or neutral nature.

### 3.2. Taxonomic Classification and Phylogenetic Analysis of SmTCPs Genes

TCPs from plant species phylogenetically related to *S. muricatum*, such as potato and tomato, and the model plant *A. thaliana* were selected (Figure 2) to classify the SmTCPs accurately. Subsequently, a total of 126 proteins, including all SmTCPs, SlTCPs, StTCPs, and AtTCPs, were subjected to multiple sequence alignment followed by the construction of a phylogenetic tree. Compared to *A. thaliana*, TCP members of *S. lycopersicum*, *S. tuberosum*, and *S. muricatum* were more closely related, since they belong to the same family and genus. In addition, the number of TCPs in *S. muricatum* is higher than those in *S*. *lycopersicum*, *S. tuberosum*, and *A. thaliana*.

Phylogenetic analysis revealed that the CYC/TB1 subcluster contained 16 SmTCP proteins, with 3 AtTCPs, 3 StTCPs, and 6 SlTCPs. The CIN subcluster comprised 11 SmTCP proteins, with 8 AtTCPs, 10 StTCP10s, and 11 SlTCPs. In Class I, 14 SmTCPs, 13 AtTCPs, 18 StTCPs, and 13 SlTCPs were clustered based on their phylogenetic relationships. Furthermore, the AtTCPs with similar functions were generally clustered in the same class of subfamilies. *AtTCP1*, *AtTCP12*, and *AtTCP18* in the CYC/TB1 class regulate lateral branch formation and flower development [41]. *AtTCP8*, *AtTCP14*, *AtTCP15*, *AtTCP22*, and *AtTCP23*, belonging to the Class I subfamily, play a crucial role in regulating leaf development through the cell cycle and the gene network maintained by stem apical meristem [42,43]. Genes in the CIN subcluster, such as *AtTCP2*, are regulated by miRNA319 [44,45], which acts as a negative regulator of cell proliferation and controls leaf margin growth. Therefore, we speculate that SmTCPs clustered in the same subfamily might have similar functions.

### 3.3. Structural and Conserved Sequence Analyses of SmTCPs

**Figure 3:**
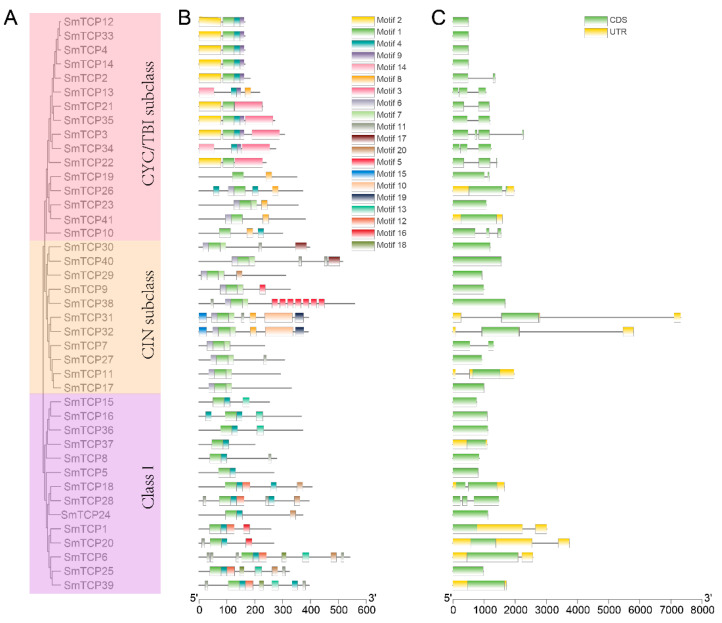
Phylogenetic tree, conserved motifs, and gene structure analyses of SmTCPs. (**A**): Phylogenetic tree. (**B**): Conserved motif. The colored boxes on the right denote motifs 1–20. (**C**): Gene structure. The yellow boxes, black lines, and green boxes represent UTR, introns, and CDS, respectively.

Each SmTCP family member has its unique sequence and structural characteristics. Conserved motifs were predicted in SmTCPs to understand their evolutionary relationships and structural diversity (Figure 3b). In total, 20 conserved motifs were detected in 41 SmTCPs (Figure S2). All the SmTCP proteins contained motif 1, except for *SmTCP13* and *SmTCP34*. Therefore, motif 1 is considered to be crucial for the members of this TCP family to perform their functions. Most SmTCP proteins in the same subfamily shared a similar motif composition. The CYC/TB1 subcluster contained motif 2, the CIN subcluster contained motifs 1 and 6, and the Class I subfamily contained motifs 1 and 4. Some motifs are unique to individual subfamilies, which demonstrates their link with a specific function. While motifs 2, 3, 14, and 9 were present in the CYC/TB1 subcluster, motifs 15, 10, 19, and 17 were specific to the CIN subcluster. In addition, motifs 12, 16, and 18 were present only in Class I proteins. The occurrence of these motifs might be attributed to their functional specificity in SmTCPs. Overall, motifs in the same subcluster were highly similar in structure, which is consistent with the phylogenetic relationship among these genes.

The cDNA sequence of each TCP was aligned with the genomic sequence to obtain its conserved motif and gene structure for understanding the evolutionary relationships and sequence characteristics of SmTCPs. As depicted in Figure 3C, most SmTCPs genes have a simple structure. While 30 (73.17%) of the 41 SmTCPs genes contained only CDS, 11 (26.83%) genes contained untranslated regions (UTRs). Of these, six genes (14.63%) had two UTRs, three genes (7.32%) had three UTRs, and the remaining two genes (4.88%) had four UTRs. A comparison of the genetic structure and phylogenetic tree of SmTCPs revealed that most members of the same subfamily share a similar gene structure in terms of length and number of exons. In addition, the number of exons in SmTCPs genes varied within the range of one to four. These results suggest that the gene structure of SmTCPs is simpler compared to *A. thaliana* [46].

### 3.4. Chromosome Distribution of SmTCPs

The chromosomal distribution of TCP genes in *S. muricatum* was heterogeneous. We observed that 40 of the 41 TCP genes were localized to 12 chromosomes in *S. muricatum* (Figure 1). Of these, Chr9, Chr10, Chr11 and Chr12 each contain only one SmTCP, while Chr1 and Chr8 are the most abundant, containing six TCPs, respectively. Next, five TCPs were located on Chr2, three TCPs on Chr4, two TCPs on Chr5 and Chr7, and four TCPs on Chr6. The highest number of TCP genes (eight) was predicted on Chr3. In addition, most TCP genes were located in the proximal or distal regions of the chromosomes.

### 3.5. Gene Duplication Analysis and Collinear Analysis 

**Figure 4:**
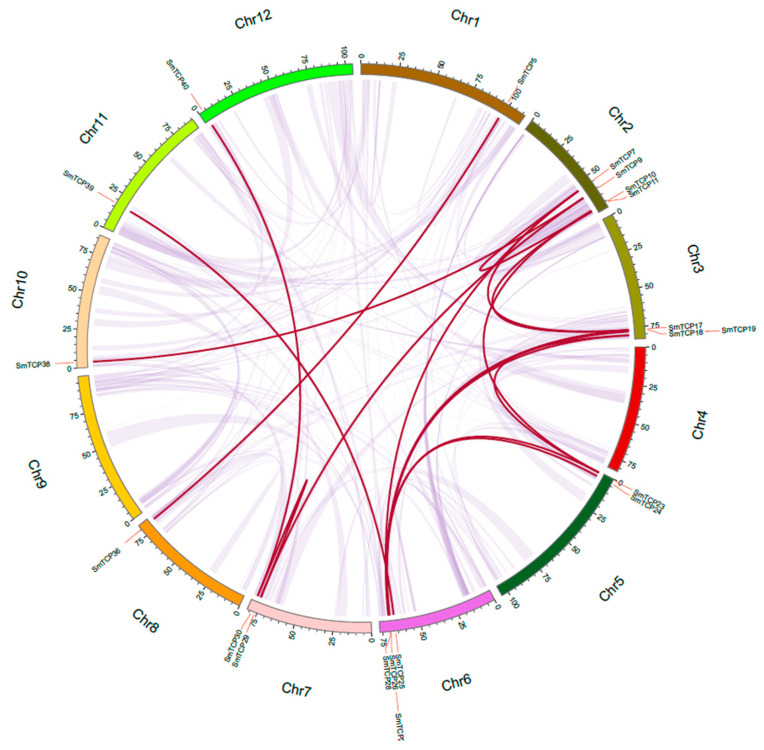
Statistical analysis of genomic tandem repeats in *S. muricatum*. Note: 17 SmTCP duplication pairs are linked with red lines. Scale bar marked on the chromosome indicating chromosome lengths (Mb).

Duplications of genomic sequences, such as whole-genome duplications (WGD), segmental duplications, and tandem duplications, provide a genetic cue for evolution [47]. Analysis of gene duplication events for the TCP gene family was performed by BLASTP and MCScanX (Figure 4). Tandem repeat sequence pairs were found on all chromosomes, except for Chr4 and Chr9, involving 17 tandem repeat sequences and 17 SmTCP gene pairs (*SmTCP7*–*SmTCP27*, *SmTCP17*–*SmTCP27*, *SmTCP30*–*SmTCP40*, *SmTCP25*–*SmTCP39*, *SmTCP9*–*SmTCP38*, *SmTCP23*–*SmTCP26*, *SmTCP19*–*SmTCP26*, *SmTCP18*–*SmTCP28*, *SmTCP7*–*SmTCP11*, *SmTCP7*–*SmTCP17*, *SmTCP11*–*SmTCP17*, *SmTCP10*–*SmTCP23*, *SmTCP19*–*SmTCP23*, *SmTCP29*–*SmTCP30*, *SmTCP9*–*SmTCP29*, *SmTCP24*–*SmTCP28*, and *SmTCP5*–*SmTCP36*). Seven SmTCPs were detected on Chr2, the maximum number among all the chromosomes.

**Figure 5:**
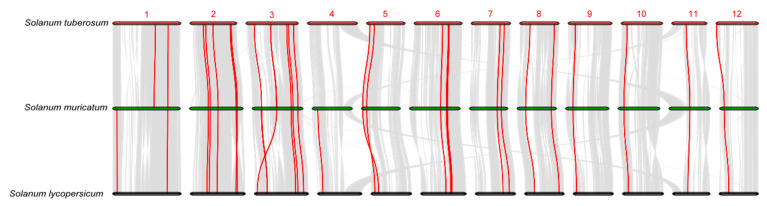
Chromosomal distribution and collinearity analysis of TCP genes in *S. lycopersicum*, *S. tuberosum*, and *S. muricatum*. Note: Gray lines in the background represent the collinear blocks within the genomes of *S. muricatum* and other *Solanum* species, while the red lines show the collinear TCP gene pairs.

To further infer the phylogenetic mechanisms of SmTCPs, a gene collinearity analysis of *S. muricatum*, associated with *S. lycopersicum* and *S. tuberosum*, was constructed. In total, 30 and 31 TCP family members were identified in *S. lycopersicum* and *S. tuberosum*, respectively (Figure 5). These results indicate that 26 TCP genes had collinearity between *S. lycopersicum* and *S. muricatum*. In addition, 25 TCP genes showed collinearity between *S. tuberosum* and *S. muricatum*. The number of synteny events of SmTCPs with *S. lycopersicum* and *S. tuberosum* differed by only one, which may be related to the fact that they belong to the same family and are evolutionarily closely related. Most TCP genes from *S. muricatum* shared homology with those in *S. lycopersicum* and *S. tuberosum*. Co-linked genes between closely related species contain a large amount of homologous information. In addition, comparative collinearity between different species can effectively identify homology and function, suggesting that their common genes may have similar functions [48,49].

### 3.6. Response of SmTCPs Expression with Different Light Treatments

**Figure 6:**
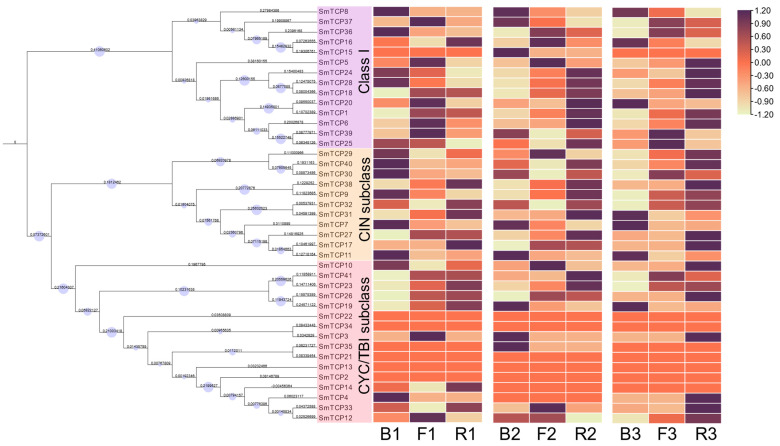
Heat map and hierarchical clustering of the SmTCP genes under each lighting treatment. Note: blue light (B), red light (R), full spectra (F) and three stages (B1–R1, B2–R2, and B3–R3).

To identify functionally relevant candidate light-response genes, the expression of 41 TCPs was obtained from the RNA-seq to elucidate the regulatory mechanisms and mode of action of SmTCPs. We divided all TCP genes into three groups by the subclass. Hierarchical clustering was performed according to the expression of 41 genes and revealed that expression clusters with functionally similar molecules. As a result, combined clustering reveals that genes with similar functions might exhibit a common expression pattern under a certain experimental condition. The identified 41 TCPs were differentially expressed at different levels in response to light (Figure 6). TCPs in the Class I and CIN subclusters were highly expressed under R treatment in the second and third stages. In the CYC/TBI subclass, most of the genes are low or not expressed, with no obvious pattern. 

### 3.7. Effect of Red and Blue Light on Differential Expression Genes in S. muricatum 

To genetically characterize the key differential expression genes’ responses to light, DETCPs were further analyzed under two different light treatments: blue light (B) and red light(R), at three stages (B1–R1, B2–R2, and B3–R3), respectively, to illustrate the molecular mechanism underlying their light response. Eight significant DETCPs were classified into three subclusters (Table 2), including *SmTCP19*, *SmTCP26*, and *SmTCP23* in the CYC/TBI subclass, *SmTCP40* and *SmTCP11* in the subclass CIN, and *SmTCP24*, *SmTCP16*, and *SmTCP28* in Class I. Among the eight DETCPs, the expression of SmTCPs in the CYC/TBI subcluster was higher in R than in B and F. However, the expression level of SmTCPs in the CIN subcluster was higher in B. On the other hand, there was no distinct expression pattern observed in Class I. In addition, *SmTCP16* was highly expressed in B, whereas *SmTCP24* and *SmTCP28* were highly expressed in R.

**Figure 7:**
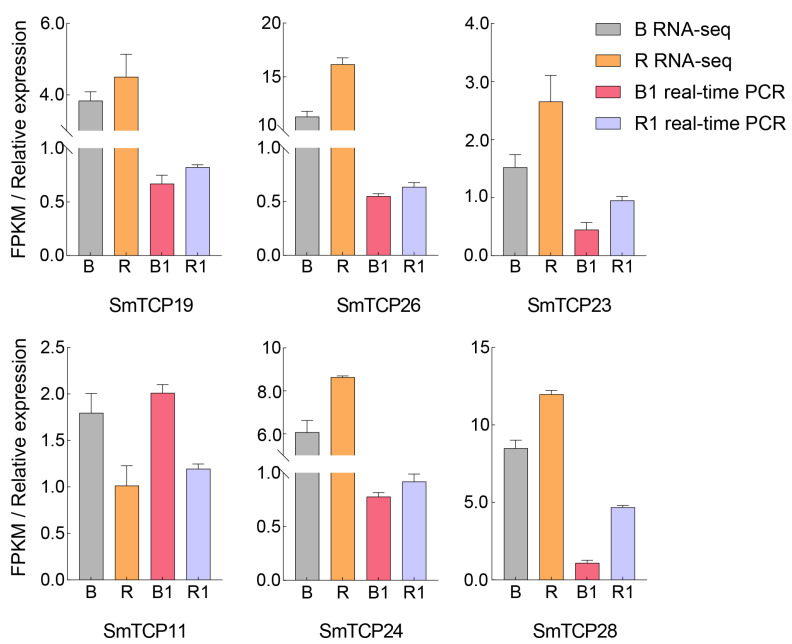
qRT-PCR validation of DETCP transcripts.

To verify the accuracy of the quantitative results of the RNA-seq data, RNA was extracted from the bud base of cutting seedlings of *S. muricatum* treated with different light qualities in B, R and F light treatment for 10 d, 20 d, and 30 d, and six DETCPs in three subclusters (CYC/TBI, CIN, and Class 1) were randomly selected with reference to expression profiling. qRT-PCR was further performed to verify the six DETCPs and determine their expression amounts (Table S2). By comparing the FPKM values in the RNA-seq data (Figure 7), it was found that the trends of the fluorescence quantitative relative expression of the six DETCPs and the corresponding FPKM values were basically consistent.

### 3.8. Interaction Network of S. muricatum DETCPs

**Figure 8:**
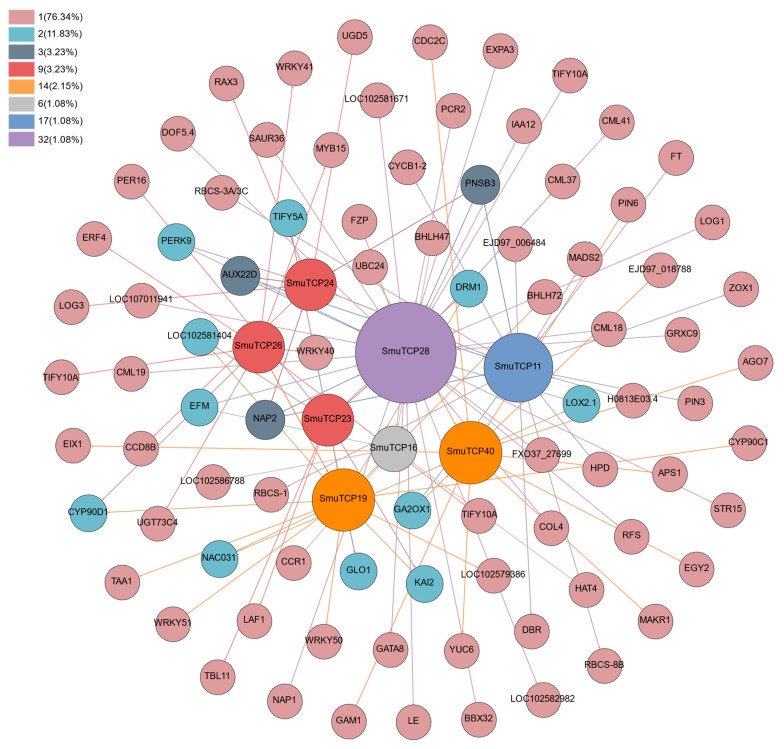
Interaction network between DETCPs and their upstream and downstream regulatory genes in *S. muricatum*. Note: Different colors represent different degrees of connectivity, the percentages in parentheses indicate the percentage of that degree.

To further clarify the light regulation mechanism of TCP, the regulatory network of eight *S. muricatum* DETCPs was analyzed with RNA-seq data. (Figure 8; Table S3). *SmTCP28* is correlated with the largest number of genes in this panel. The regulatory genes identified in the CYC/TBI subcluster involved multiple regulatory pathways including *TIFY5A* (AT1G30135), *TIFY10A* (AT1G19180), *NAC031*, *KAI2* (KARRIKIN INSENSITIVE2), *BHLH72*, *WRKY51*, *ERF4* (ethylene-responsive element binding factors), *CYP90D1* (AT3G13730.1), *MYB15*, and *SmTCP28*. *KAI2*, *ERF4*, and *CYP90D1* were involved in hormone metabolic pathways, such as strigolactone, brassinolide, and ethylene. Furthermore, a large number of hormone-related genes were identified in the CIN subcluster and the Class 1 subcluster. The gene regulating *SmTCP40* was primarily *LOX2.1* (AT3G45140). Growth hormone synthesis- (*YUC6* and *AUX22D*) and perception-related (*PIN6*) genes, gibberellins (*GA2OX* (GA2-oxidase)), and cytokinin (*CYCB1-2* (AT4G37490)) were also observed to be involved in the regulatory mechanism of TCP genes. Light-regulated genes (ribulose-1,5-bisphosphate carboxylase/oxygenase (*RBCS*), *PNSB3* (PHOTOSYNTHETIC NDH SUBCOMPLEX B 3), *EGY2* (ETHYLENE-DEPENDENT GRAVITROPISM-DEFICIENT AND YELLOW-GREEN-LIKE 2), and *RAX3* (MYB84, morphological regulator)) were also identified from the interactions with other genes. We also predicted cis-acting elements based on the promoter sequences of TCP family genes. The results show that the identified cis elements were involved in light response, abiotic stress, circadian control and hormone signaling (Figure S3; Table S4). It appears that TCP family genes regulate a wide range of physiological functions. In accordance with previous reports, this study identified several functional genes that are regulated by TCPs.

### 3.9. Response of AtTCP Expression with Different Light Treatment and Co-expression Analysis

**Figure 9:**
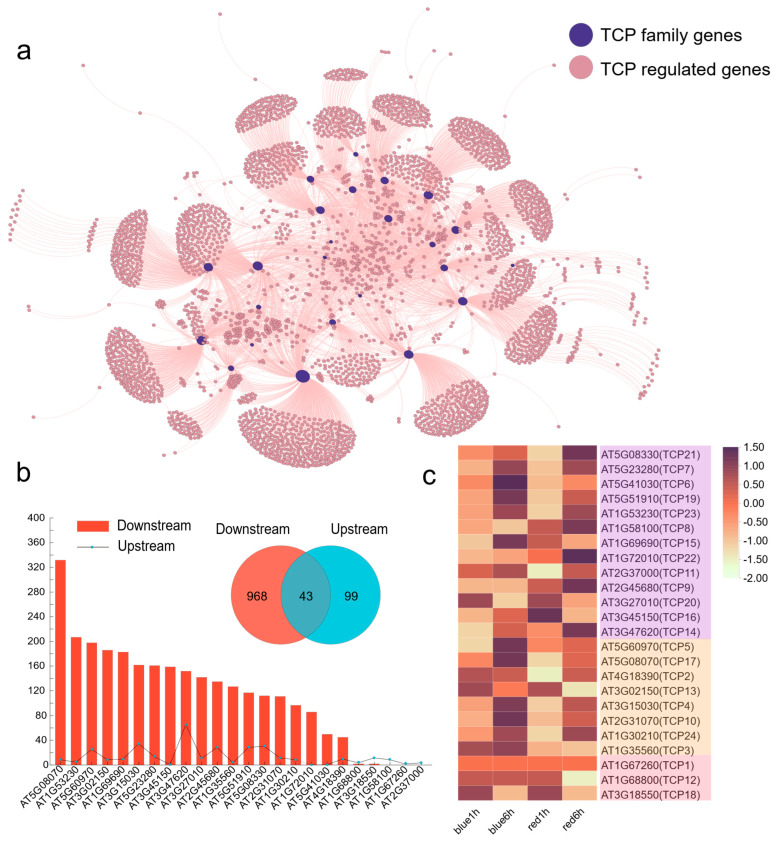
Interaction network between TCP family genes and their upstream and downstream regulatory genes in *A. thaliana*. (**a**): the network was constructed using gephi software; (**b**): the number of upstream and downstream genes for each TCP in the network; specific and common genes between downstream and upstream genes in the network; (**c**): expression profile of AtTCPs in *Arabidopsis* after red- and blue-light irradiation periods.

As a model crop, the function of AtTCPs has been widely verified. Therefore, more regulatory mechanisms of SmTCPs can be further clarified through the comparison of homologous genes. Co-expression analysis is a robust method for predicting gene function and its regulatory mechanisms. We constructed an interaction network of the TCPs and their target genes in *A. thaliana* (Figure 9a; Table S5). The regulatory network consisted of 3085 gene pairs containing 1011 downstream genes (regulated by TCP family genes) and 143 upstream genes (regulated TCP family genes) (Figure 9b; Table S6). Among them, the number of genes regulated by different TCPs varied significantly. The largest number of downstream genes (332) was found to be associated with *AT5G08070*, followed by *AT1G53230* (207) and *AT5G60970* (198). However, three TCP family genes had no downstream genes, including *AT1G58100*, *AT1G67260*, and *AT2G37000*. Similarly, *AT3G47620* had the highest number of upstream genes (65), whereas no upstream genes were found for *AT1G72010*, *AT3G45150*, and *AT5G41030*. Additionally, 43 genes were found to belong to both downstream and upstream genes, indicating that these genes were likely feedback-regulated (Table S7).

**Figure 10:**
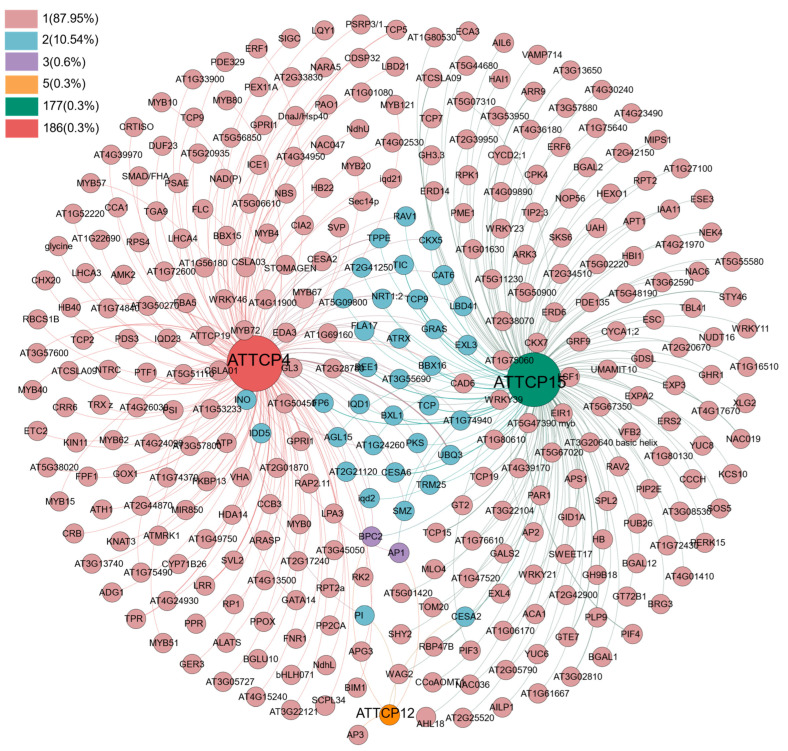
Gene regulatory network of *AtTCP12*, *AtTCP4* and *AtTCP15*. Note: Different colors represent different degrees of connectivity, the percentages in parentheses indicate the percentage of that degree.

The expression of TCP genes in *A. thaliana* was investigated under different light treatments. *AtTCP12*, *AtTCP4*, and *AtTCP15* showed reduced expression in R and elevated expression in B after 6 h of light treatment (Figure 9c; Table S8). The expression of *AtTCP12* and *AtTCP15* was the opposite compared to that in *S. muricatum*. *AtTCP12* (BRC2), a TF regulating lateral branch development, is synthesized in the roots and acts on branches to inhibit lateral bud genesis [50]. In addition, *AtTCP15* plays a crucial role in regulating endoreduplication during *A. thaliana* development [51] and affects the growth of petioles, pedicels, and anther filaments [52]. However, the specific regulatory mechanisms of these genes and their targets need to be investigated further. Homologous genes of SmTCPs were identified by aligning the phylogenetic trees constructed in *S. muricatum*, *S. lycopersicum*, and *A. thaliana* (Figure 2; Table 2). Subsequently, the functions and regulatory mechanisms of their DETCPs in different crops (*Arabidopsis*) were detected. Three *A. thaliana* homologous TCPs (*AtTCP12*, *AtTCP4*, and *AtTCP15*) were identified in CYC/TBI, CIN, and Class I subclusters and subjected to gene regulatory network analysis (Figure 10; Table S9). *AtTCP12* regulated the lower number of genes compared to *AtTCP4* and *AtTCP15*. *CESA2* and *BPC2* may function downstream of *AtTCP12*, whereas *PI* (PISTILLATA), *AP3* (APETALA3), and *AP1* (APETALA1) belong to their upstream pathway. *AtTCP4*-related regulators are relatively abundant. Among the downstream regulators of *AtTCP4*, *PSAE-1* (Prairie State Achievement Exam), *LHCA4* (light-harvesting complexes), and *LHCA3* were involved in the light regulation, which accounted for the differential expression of *SmTCP40* among different light treatments in *S. muricatum*. In addition to the light regulation factors, numerous hormone regulation-related genes such as *ERF1*, *CYP71B26*, *CKX5* (Cytokinin Oxidase 5), *BEE1* (Brassinolide Enhanced Expression1) and *CRTISO* (Carotenoid Isomerase) were also identified downstream of *AtTCP4*. The complex regulatory mechanisms of *AtTCP4* also include *CSLA01* (Cellulose Synthase-like), *FPF1* (Flowering Promoting Factor1), and *GRAS* apical regulatory factor. Further exploration of the regulatory mechanism of *AtTCP15* revealed that its downstream pathway was also complex, involving *NPH3* (NON-PHOTOTROPIC HYPOCOTYL 3; light regulatory factor), *RPT2* (Root Phototropism; light regulatory factor), and *PAR1-Phy* (Protease-activated receptors-PHYTOCHROME; red light receptor). The *SmTCP24* was highly expressed in R (Table 2). Multiple genes related to the hormone regulatory pathway were also identified downstream of *AtTCP15*, including *BEE1*, *EIR1*, *SHY2* (SHORT HYPOCOTYL 2), *ERS2*, and *ARR9* (*A. thaliana* response regulators), as well as regulators related to brassinolide, growth hormone, and ethylene. Genes associated with the regulation of rapeseed endosperm, auxin, and ethylene were also identified in our study. The upstream regulators contained multiple phytochrome interaction factors, such as *PIF3* and *PIF4*, and the regulatory factors of miR172-SMZ (Sulfamethoxazole). 

### 3.10. Comparative Analysis of TCP Regulatory Genes Annotation between ARABIDOPSIS and S. muricatum

To conduct functional annotation, the TCP regulatory genes were compared with GO annotation in the *A. thaliana* and *S. muricatum.* Based on our network analysis, genes with different expression profiles are highly interconnected. Gene ontology biological process analysis revealed the enrichment of genes associated with cellular processes, developmental processes and metabolic processes in three orthologous genes across the two species. Within this classification, the other two functional annotations (Molecular Function and Cellular Component) identified for three genes were also the same (Figure S4). The functions of TCP-regulated genes in *S. muricatum* are particularly similar to those of *A. thaliana*, and among the three homologous genes, the GO pathways annotated by SmTCP40 and SmTCP24 were retrieved in homologous GO terms in *A. thaliana*, and the pathways annotated by SmTCP23 differed in only one pathway from *A. thaliana*. That said, many pathways regulated by TCPs were consistent, despite the low number of differentially regulated genes in *S. muricatum*. The emergence of conserved signaling pathways and regulatory mechanisms suggests that the signaling pathways downstream of TCPs may be conserved to some extent between *A. thaliana* and *S. muricatum*. 

## 4. Discussion

The gene function analysis of *S. muricatum*, which belongs to the genus *Solanum*, has been limited to a few reports. The TCP gene family plays a crucial role in various crops′ morphological evolution and development. This gene family analysis in *S. muricatum* was facilitated after the decoding of the *S. muricatum* genome. The purpose of this study was to identify SmTCPs and to analyze their evolutionary relationship and functional characteristics. Forty-one SmTCP proteins with TCP structural domains were identified, a number higher than the numbers in *A. thaliana*, *S. lycopersicum* and *S. tuberosum*. The genome of *S. muricatum* is ~1.20 Gb [53], whereas it is 133.75 Mb [54], 760 Mb [55], and 835.1 Mb (A6–26) to 1.71 Gb for *Arabidopsis*, *S. lycopersicum* and *S. tuberosum*, respectively. However, polyploidy is found in *S. tuberosum* [56]. It is common to find multiple copies of many genes in polyploid plants due to polyploidization or WGD events [57]. Though the total numbers of gene family members do not scale linearly to the genome sizes, the proportion of gene family members is correlated with the genome size [58,59]. Based on the number of genes and evolutionary relationships among the identified TCP gene families, SmTCPs were more numerous than AtTCPs, StTCPs, and SlTCPs, but the evolutionary classification was similar to that for other crops, suggesting that *S. muricatum* may have undergone gene duplication and multiple gene copy retention events during evolution [60]. The evolution of *S. muricatum* has rarely been studied, and the evolutionary relationship will be supplemented when the high-quality genome of *S. muricatum* is resolved.

**Figure 11:**
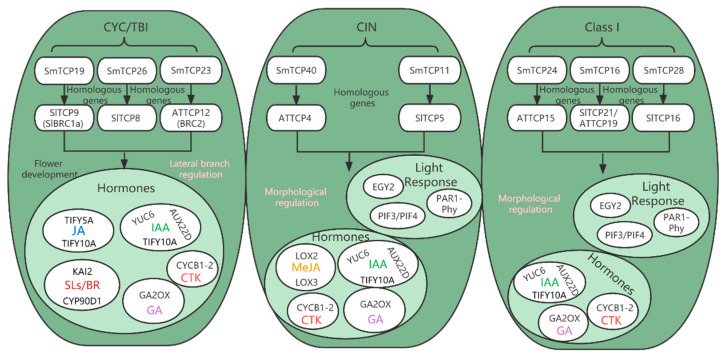
Summary of regulatory mechanisms of CYC/TBI, CIN, Class I.

Further investigations of the TCP gene family were conducted to determine how it responds to light (Figure 11). According to our RNA-seq and qRT-PCR results, SmTCPs respond to different light treatments. Eight TCPs were identified in CYC/TBI, CIN and Class I. Among them, CYC/TBI contains three differential genes, *SmTCP19* (*SlTCP9*-*SlBRC1a*), *SmTCP26 (SlTCP8)* and *SmTCP19* (*AtTCP12*-*BRC2*). Their regulatory genes were primarily *TIFY5A* and *TIFY10A*. *TTIFY10A* is not only a jasmonic acid-induced gene, but also an early auxin response gene [61,62]. The regulatory genes *KAI2* and *CYP90D1* are involved in hormone-related metabolic pathways, such as strigolactone and brassinolide [63,64]. *KAI2* can bind to and be activated by D14 protein of strigolactone. It can regulate seedling morphogenesis by affecting the auxin transport system [65]. Comparing the *AtTCP12* interaction network with the two sub-clusters of CIN and Class I showed that *AtTCP12* had the fewest genes and was relatively conserved (Figure 9). CYC-TB1 is associated with lateral branching regulation and flower development [66]. However, linking Gene Regulatory Networks with the *AtTCP12* identified in this study, *PI*, *AP3*, and *AP1* were all associated with flower development [67]. In addition, we did not detect any genes that regulate lateral branching in *A. thaliana*. This may be caused by the identification of target genes of the AtTCPs using the Integrated Gene Regulatory Network (iGRN) database with a score ≥0.80, resulting in the filtration of less significantly expressed genes. *SmTCP19* (SlBRC1a), *SlTCP8*, and *AtTCP12* (BRC2) in the CYC/TBI subcluster of *S. muricatum* interact with auxin and strigolactone in apical dominance and lateral branching regulation [68,69], explaining the mechanism of CYC/TBI. According to our hypothesis, SmTCPs are involved in lateral branch genesis and apical dominance in *S. muricatum* under different light quality treatments. Our previous studies on *S. muricatum* cuttings also confirmed this result.

The regulatory gene for *SmTCP40* in both CIN and Class I subclusters was *LOX2.1*. Similar to *LOX3*, *LOX2* is stimulated by methyl jasmonate [70], an analog of jasmonic acid. JA has been proven to be one of the key regulators of phytochrome signaling [71]. The regulatory mechanism of *AtTCP4* and *AtTCP15*, homologs of *SmTCP40* and *SmTCP24*, is consistent with that of *S. muricatum*. A large number of genes related to hormone regulation, such as auxin (*SHY2*), ethylene (*ERF1*), cytokinin (*CYP71B26, CKX5, ARR9*), and brassinolide (*BEE1*), were also identified downstream of *AtTCP4*, and *AtTCP15* in *A. thaliana* was similar to *S. muricatum*, in addition to light quality regulators. The complex regulatory mechanisms of *AtTCP4* and *AtTCP15* also identified *PAR1-Phy* red light receptor, whereas the *S. muricatum* homolog *SmTCP24* was highly expressed in red light (Table 2). Multiple phytochrome interaction factors such as *PIF3* and *PIF4* were identified from upstream regulatory factors. Members of the *PHYTOCHROME-INTERACTING FACTOR* (PIF) TF family play a central regulatory position in mediating the plant response to light signals [72]. Furthermore, CIN-like TCP TFs interact with PIF proteins in plant response to light during the nutritional growth phase [25]. 

In addition to studying co-expression networks, it was found that AtTCPs and SmTCPs showed inconsistent responses to blue and red light based on expression levels, which was speculated to be caused by the different sites at which gene expression was measured. The RNA-seq data from our plants were collected at the base of the lateral buds, whereas those from *Arabidopsis* were collected from the whole plant. This indicates that TCP genes are tissue-specific. However, TCPs may possess evolutionarily conserved functionality in gene regulation. A comparative analysis of signaling pathways upstream and downstream of TCPs showed that the TCP regulatory pathway is conserved among species. This confirms our initial hypothesis. Despite the overall similarities between the species, it is limited to only *Arabidopsis* and *S. muricatum*. The regulatory pathway for TCP light response in many species has not yet been established. Several pilot studies have demonstrated the extensive functional conservation of TCPs in *Rosaceae* species [73]. The results will need to be further studied to verify the initial findings. Overall, an important theoretical basis for improving *S. muricatum* quality will be provided by analyzing the biological functions of these genes.

## 5. Conclusions

In the current study, we identified and characterized 41 members of the SmTCP gene family. Evolutionary analysis suggested that the taxonomy of SmTCPs was similar to that of AtTCPs. The CYC/TB1 subcluster, CIN subcluster, and Class I subfamily contained 16, 11, and 14 SmTCPs, respectively. The SmTCPs and the mechanisms of their responses to red and blue light were classified using phylogenetic and gene expression regulation. Thus, the TCP genes′ responses to different light qualities (red and blue) were clarified. In addition to the response to light quality, functional regulation was mostly enriched with hormone signaling response, hypocotyl elongation, and lateral branch genesis. It is therefore essential to study the response of SmTCPs to light processing, and our findings provide a basis for further analysis of the TCP gene family in other *Solanaceae* crops and broaden the functional insights into TCP genes regarding the light response.

## Figures and Tables

**Table 1:** Information on members of TCP transcription factor family in *S. muricatum*.

Gene ID	TCP Number	Chromosome Location	Amino Acid	Molecular Weight (Da)	Isoelectric Point
Smu01G002900.1	SmTCP1	Chr01:3332723-3335734	258	26,738.7	9.25
Smu01G010770.1	SmTCP2	Chr01:27066460-27067813	183	21,100.8	10.14
Smu01G010850.1	SmTCP3	Chr01:27800921-27803184	307	35,322.1	10.71
Smu01G012690.1	SmTCP4	Chr01:43848293-43848790	165	19,012.3	10.38
Smu01G028040.1	SmTCP5	Chr01:98110326-98111135	269	29,026.1	11
Smu01G037260.1	SmTCP6	Chr01:105533447-105536012	541	58,109.5	7.42
Smu02G012810.1	SmTCP7	Chr02:54705676-54706978	236	26,613	9.71
Smu02G014130.1	SmTCP8	Chr02:55729354-55730193	279	30,015.3	8.73
Smu02G019380.1	SmTCP9	Chr02:60587194-60588180	328	37,236.6	5.24
Smu02G031600.1	SmTCP10	Chr02:70759903-70761444	300	33,948.8	8.87
Smu02G032390.1	SmTCP11	Chr02:71403226-71405184	292	32,605.8	7.17
Smu03G006140.1	SmTCP12	Chr03:13497696-13498193	165	18,902.3	10.51
Smu03G006260.1	SmTCP13	Chr03:14258604-14259658	218	25,333.8	11.22
Smu03G006480.1	SmTCP14	Chr03:16602979-16603476	165	18,863.3	10.54
Smu03G016390.1	SmTCP15	Chr03:68102671-68103432	253	27,101.6	8.47
Smu03G016550.1	SmTCP16	Chr03:68342489-68343592	367	38,983.3	4.82
Smu03G026310.1	SmTCP17	Chr03:76801657-76802652	331	37,555.6	6.08
Smu03G027550.1	SmTCP18	Chr03:77806134-77807784	406	42,963.9	7.47
Smu03G030930.1	SmTCP19	Chr03:80379818-80380979	351	39,923.1	9.4
Smu04G003880.1	SmTCP20	Chr04:4042925 -4046662	268	28,082.3	10.39
Smu04G011520.1	SmTCP21	Chr04:28185097-28186262	229	26,440.1	11.03
Smu04G012560.1	SmTCP22	Chr04:38220364-38221776	241	27,598.3	10.75
Smu05G000850.1	SmTCP23	Chr05:593333-594403	356	40,793.1	9.44
Smu05G003500.1	SmTCP24	Chr05:3280292-3281413	373	41,513.7	6.95
Smu06G021110.1	SmTCP25	Chr06:66811270-66812244	324	34,685	7.47
Smu06G024960.1	SmTCP26	Chr06:69716855-69718816	372	41,736.1	7.93
Smu06G025220.1	SmTCP27	Chr06:69948309-69949232	307	34,126.2	9.47
Smu06G025710.1	SmTCP28	Chr06:70332967-70334440	395	43,032.7	6.77
Smu07G018850.1	SmTCP29	Chr07:76470532-76471467	311	35,168.5	7.06
Smu07G021660.1	SmTCP30	Chr07:78741136-78742329	397	43,287.3	6.77
Smu08G005820.1	SmTCP31	Chr08:7958152-7965853	393	43,909.8	6.76
Smu08G005850.1	SmTCP32	Chr08:8116621-8122423	392	43,764.5	6.2
Smu08G008280.1	SmTCP33	Chr08:30519308-30519805	165	18,777.1	10.39
Smu08G008340.1	SmTCP34	Chr08:30990318-30991540	275	31,489.4	10.2
Smu08G008390.1	SmTCP35	Chr08:31805011-31806191	272	31,258.4	10.6
Smu08G023240.1	SmTCP36	Chr08:80179377-80180495	372	39,709.2	7.28
Smu09G005030.1	SmTCP37	Chr09:6222598-6223693	201	21,337	8.5
Smu10G003890.1	SmTCP38	Chr10:3619195-3620871	558	63,952.9	7.1
Smu11G014110.1	SmTCP39	Chr11:17567466-17569182	395	42,372.6	8.57
Smu12G003120.1	SmTCP40	Chr12:2972387-2973937	516	57,154.9	7.53
Smuptg000412lG000050.1	SmTCP41	ptg000412l:62921-64509	381	43,577.7	8.68

**Table 2:** Summary of significant DETCPs expressed in different light treatments and homologous genes of *S. muricatum*.

SmTCP	Classification	B_fpkm	F_fpkm	R_fpkm	*P*-Value	FDR	Homologous Genes	Homologous Encoding Genes
SmTCP19	CYC/TBI	3.84	4.04	4.51	3.11 × 10^−8^	1.92 × 10^−7^	SlTCP9	Solyc03g119770.3.1
SmTCP26	CYC/TBI	11.30	15.28	16.16	8.65 × 10^−28^	1.65 × 10^−26^	SlTCP8	Solyc06g069240.2.1
SmTCP23	CYC/TBI	1.52	2.27	2.65	0.00249590	0.008556416	ATTCP12	AT1G68800
SmTCP40	CIN	0.58	0.23	0.41	9.74 × 10^−5^	0.000387219	ATTCP4	AT3G15030
SmTCP11	CIN	1.80	0.86	1.01	0.00666871	0.025969579	SlTCP5	Solyc02g089020.2.1
SmTCP24	Class I	6.07	6.13	8.64	1.1 × 10^−13^	1.90 × 10^−12^	ATTCP15	At1G69690
SmTCP16	Class I	1.81	1.53	1.46	0.00027819	0.001131583	SlTCP21/ATTCP19	Solyc03g006800.1.1/AT5G51910
SmTCP28	Class I	8.49	10.00	11.99	1.45 × 10^−20^	1.79 × 10^−19^	SlTCP16	Solyc03g116320.3.1

## Data Availability

The data presented in this study are openly available in the NCBI (https://www.ncbi.nlm.nih.gov/sra/?term=PRJNA869486 (accessed on 14 August 2022)) and in the Supplementary Materials.

## Supplementary Materials

The following supporting information can be downloaded at https://www.mdpi.com/article/10.3390/cells12071015/s1: Figure S1: *S. muricatum* seedling phenotypes under different light treatments (30d). Figure S2: Motif 1-20 for TCP family genes. Figure S3: The cis-acting elements in the promoter of each TCP family gene in the network. Figure S4: Level 2 GO terms of homologous genes of AtTCPs and SmTCPs. Table S1: The genes and sequences of the primers used for qRT-PCR. Table S2: The Ct value plots for different SmTCP genes. Table S3: Gene interaction regulatory network of eight TCPs of *S. muricatum*. Table S4: The cis-acting elements in the promoter of SmTCP family genes in the network. Table S5: Detailed information of down- and upstream genes related to TCPs in the network of *A. thaliana*. Table S6: The edge number of down- and upstream genes related to TCPs in the network of *A. thaliana*. Table S7: The unique and common genes of down- and upstream TCPs in the network of *A. thaliana*. Table S8: The absolute expression values of TCP family genes in *A. thaliana* under blue–red light treatment from the *A. thaliana* eFP Browser. Table S9: Gene interaction regulatory network of *AtTCP12*, *AtTCP4,* and *AtTCP15*.
