# Supplementary material for: Systematic Investigation of TCP Gene Family: Genome-Wide Identification and Light-Regulated Gene Expression Analysis in Pepino (Solanum Muricatum)
Source: Cells. 2023 Mar 26;12(7):1015. doi: 10.3390/cells12071015 (PMC10093338; doi:10.3390/cells12071015)
Supplement: Supplementary file 1 [file cells-12-01015-s001.zip › supplementary Figures S1-S4.pdf]

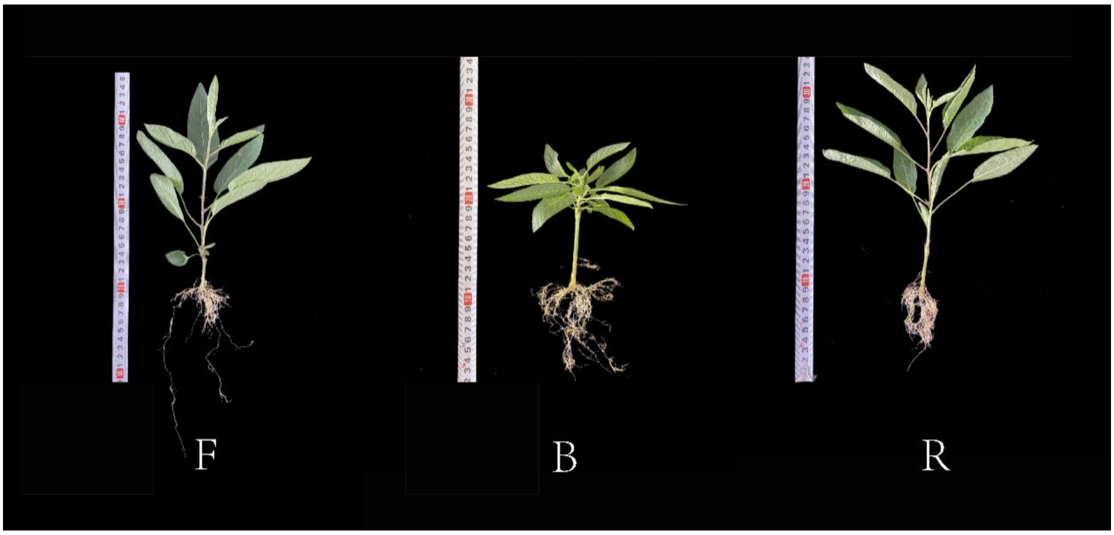

Figure S1 *S. muricatum* seedling phenotypes under different light treatments (30d)

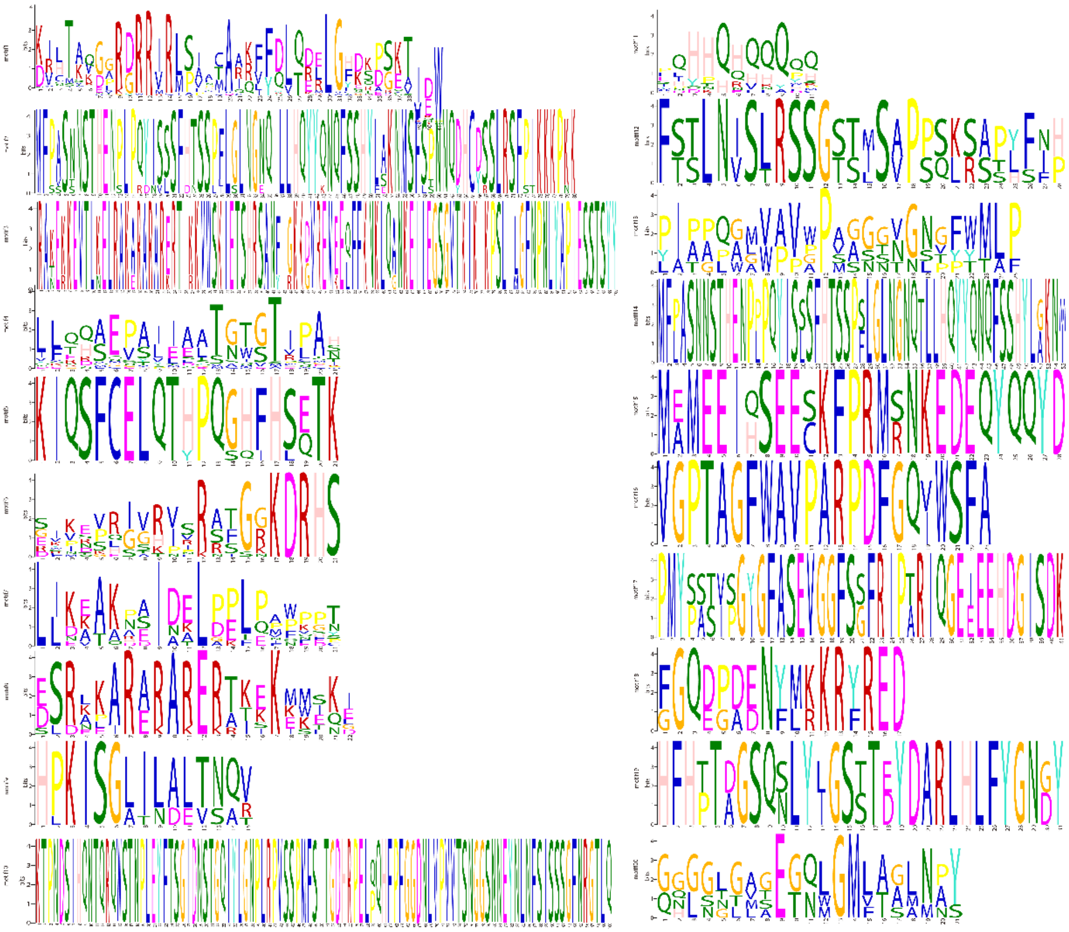

Figure S2 Motif 1-20 for TCP family genes

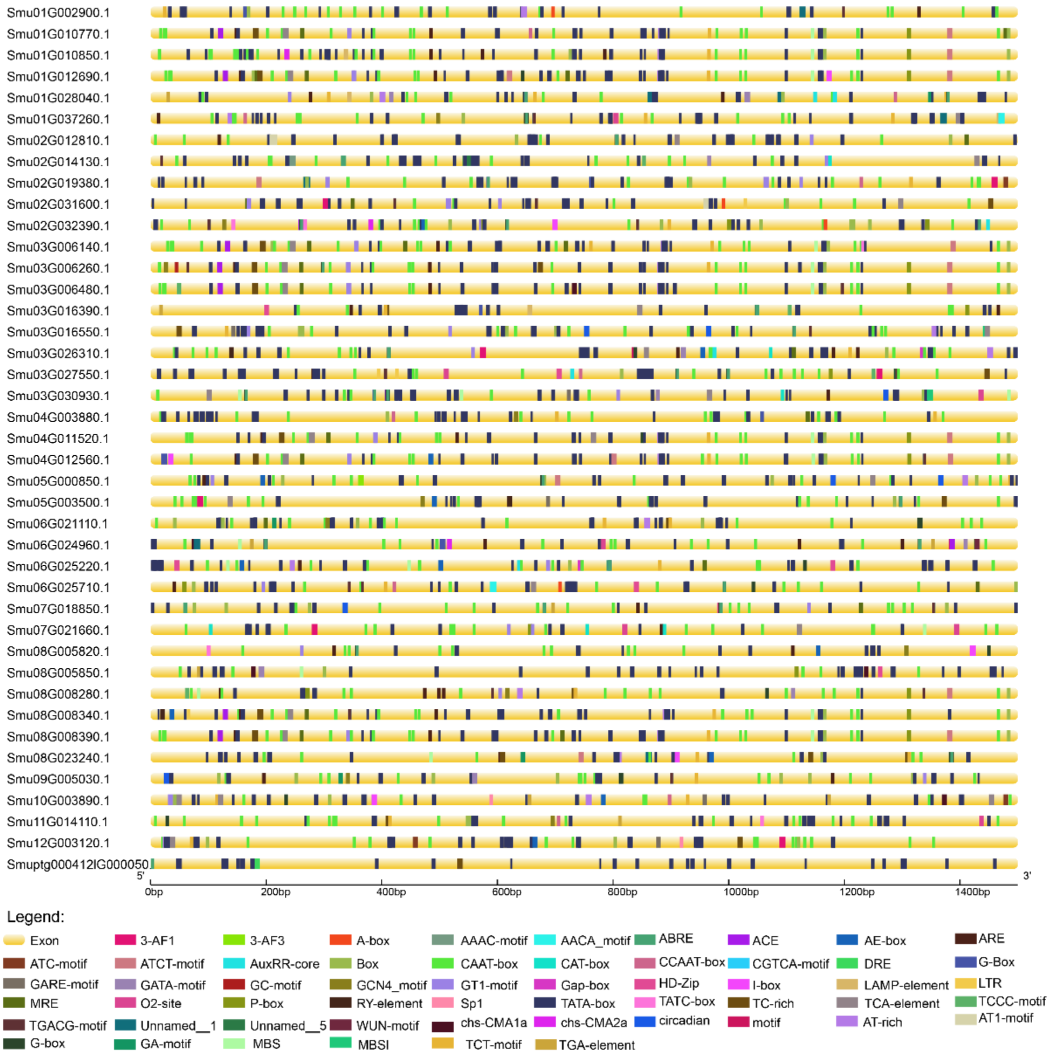

Figure S3 The cis-acting elements in the promoter of each TCP family gene in the network.

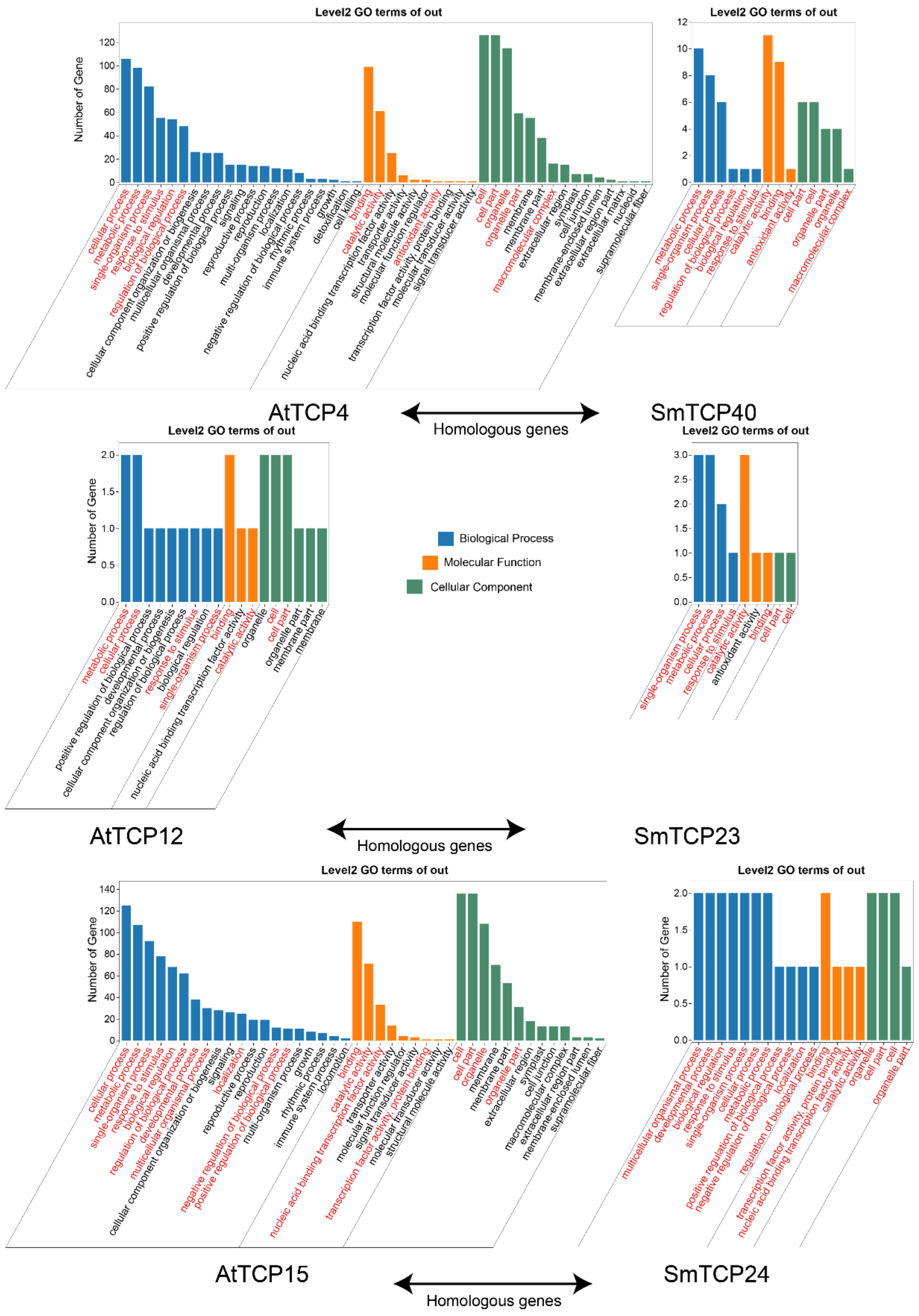

Figure S4 Level2 GO terms of homologous genes of AtTCPs and SmTCPs. Notes: Red font indicates homologous genes annotated to the same pathway.
